# Crystal structure of 7β-hy­droxy­royleanone isolated from *Taxodium ascendens* (B.)

**DOI:** 10.1107/S2056989017011987

**Published:** 2017-09-05

**Authors:** Shicheng Xu, Xinhua Ma, Ruifang Ke, Shihao Deng, Xinzhou Yang, Ping Song

**Affiliations:** a School of Pharmaceutical Sciences, South-Central University for Nationalities, Wuhan 430074, People’s Republic of China; b College of Chemistry and Life Science, Qinghai University for Nationalities, Xining 810007, People’s Republic of China

**Keywords:** crystal structure, 7β-hy­droxy­royleanone, *Taxodium ascendens*, hydrogen bonding

## Abstract

The title compound, C_20_H_28_O_4_ [systematic name: (4b*S*,8a*S*,10*S*)-3,10-dihy­droxy-2-isopropyl-4b,8,8-trimethyl-4b,5,6,7,8,8a,9,10-octa­hydro­phenanthrene-1,4-dione], is an abietane-type diterpene, which was isolated from *Taxodium ascendens* (B.). The compound crystallizes in the chiral space group *P*2_1_, but it was not possible to determine the absolute structure of the mol­ecule in the crystal by resonant scattering. The mol­ecular structure is stabilized by two intra­molecular O—H⋯O hydrogen bonds, enclosing *S*(5) and *S*(6) ring motifs. In the crystal, mol­ecules are linked by O—H⋯O and C—H⋯O hydrogen bonds, forming chains along the [010] direction. The crystal structure of the 10*R* stereoisomer of the title compound, isolated from the roots of *Premna obtusifolia* (Verbenaceae), has been reported. It crystallized in the chiral space group *P*2_1_2_1_2_1_, and the absolute structure was determined as (4b*S*,8a*S*,10*R*), by resonant scattering using Cu *K*α radiation [Razak *et al.* (2010 ▸). *Acta Cryst.* E**66**, o1566–o1567].

## Abstract 2

The title compound, 7β-hy­droxy­royleanone, an abietane-type diterpene, was isolated from *Taxodium ascendens* (B.).

## Chemical context   

*Taxodium ascendens* Brongn belongs to the Taxodiaceae species, which is native to the south-east of North America and has spread widely over southern China (Si *et al.*, 2001 ▸). Previous chemical studies of *Taxodium ascendens* (B.) have described many diterpenes, such as 6,7-de­hydro­royleanone, salvinolone and xanthoperol (Kusumoto *et al.*, 2009 ▸; Gonzalez, 2015 ▸), and the diterpenoids have attracted much attention in recent years because of their diverse biological properties (Burmistrova *et al.*, 2013 ▸; Tanaka, 2001 ▸), such as anti­bacterial (Yang *et al.*, 2001 ▸), anti­oxidant (Kolak *et al.*, 2009 ▸), anti­fungal (Topçu & Gören, 2007 ▸) and anti­cholinesterase activities (Topçu *et al.*, 2013 ▸). A detailed phytochemical investigation of a petroleum ether extract of the pollen of *Taxodium ascendens* Brongn has been carried out and a series of diterpenoids have been isolated, including the title compound, 7β-hy­droxy­royleanone. Herein, we present the crystal structure of 7β-hy­droxy­royleanone carried out in order to establish unambiguously the stereochemical features of this natural product.

## Structural commentary   

**Figure 1:**
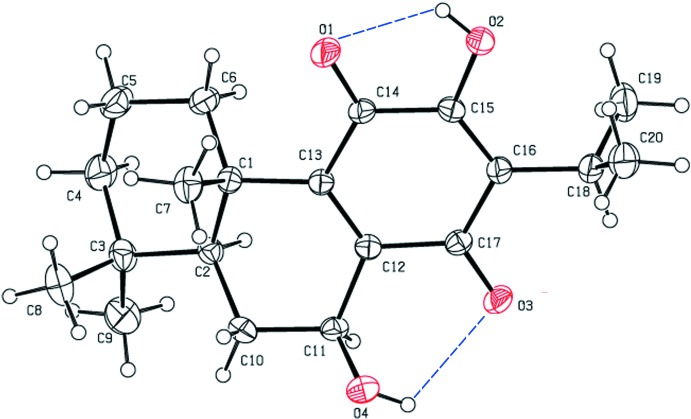
The mol­ecular structure of the title compound, with the atom labelling and 50% probability displacement ellipsoids. Intra­molecular O—H⋯O hydrogen bonds are shown as blue dashed lines (see Table 1 ▸).

The mol­ecular structure of the title compound is shown in Fig. 1 ▸. The structure contains two hy­droxy groups, located at atoms C11 and C15, two ketone groups at C14 and C17, and two double bonds, C12=C13 and C15=C16. There are two intra­molecular hydrogen bonds, *viz.* O2—H2⋯O1 and O4—H4⋯O3, which stabilize the mol­ecular conformation. Ring *A* (atoms C1–C6) has a chair conformation [puckering parameters: amplitude (*Q*) = 0.552 (2) Å, θ = 4.9 (2)° and φ = 292 (3)°], while ring *B* (C1/C2/C10–C13) has an envelope conformation, with atom C2 as the flap [puckering parameters: *Q* = 0.558 (2) Å, θ = 125.1 (2)° and φ = 256.2 (3)°]. Benzo­quinone ring *C* (C12–C17) has a screw-boat conformation [puckering parameters: *Q* = 0.097 (2) Å, θ = 66.3 (12)° and φ = 29.7 (14)°]. The mean planes of the various rings are inclined to one another in the following manner: *A*/*B* = 22.97 (10)°, *A*/*C* = 34.52 (10)° and *B*/*C* = 12.84 (9)°.
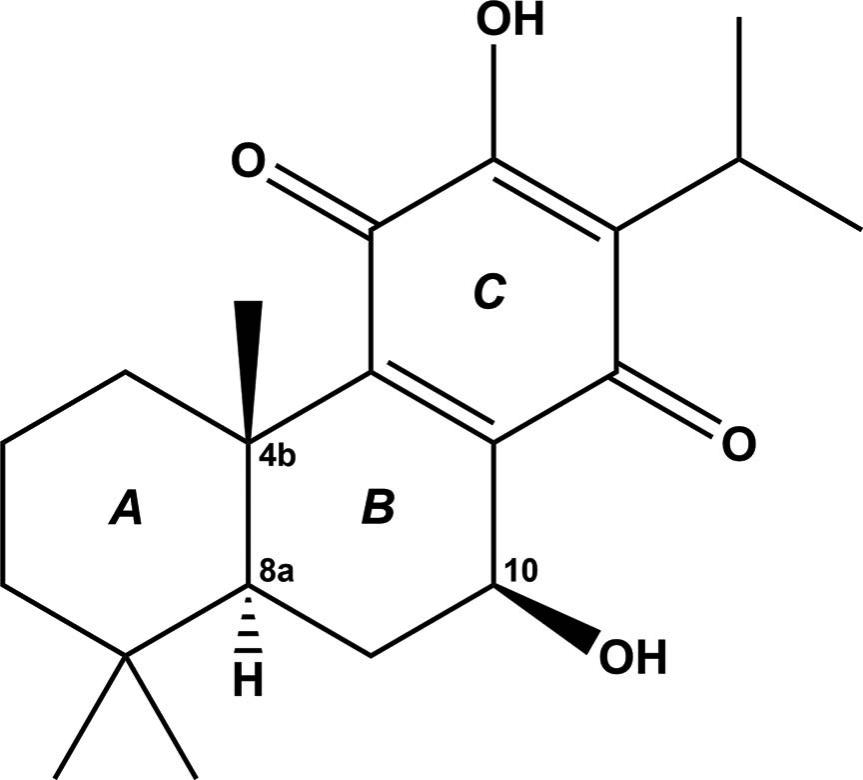


The crystal structure of the 10*R* stereoisomer of the title compound, isolated from the roots of *Premna obtusifolia* (Verbenaceae), has been reported twice (see §4, *Database survey*). It crystallized in the chiral space group *P*2_1_2_1_2_1_, and the absolute structure was determined as (4b*S*,8a*S*,10*R*) by resonant scattering using Cu *K*α radiation (Razak *et al.*, 2010 ▸). Comparing the two compounds indicates that the configuration of the three stereocentres in the title compound are (4b*S*,8a*S*,10*S*).

## Supra­molecular features   

**Figure 2:**
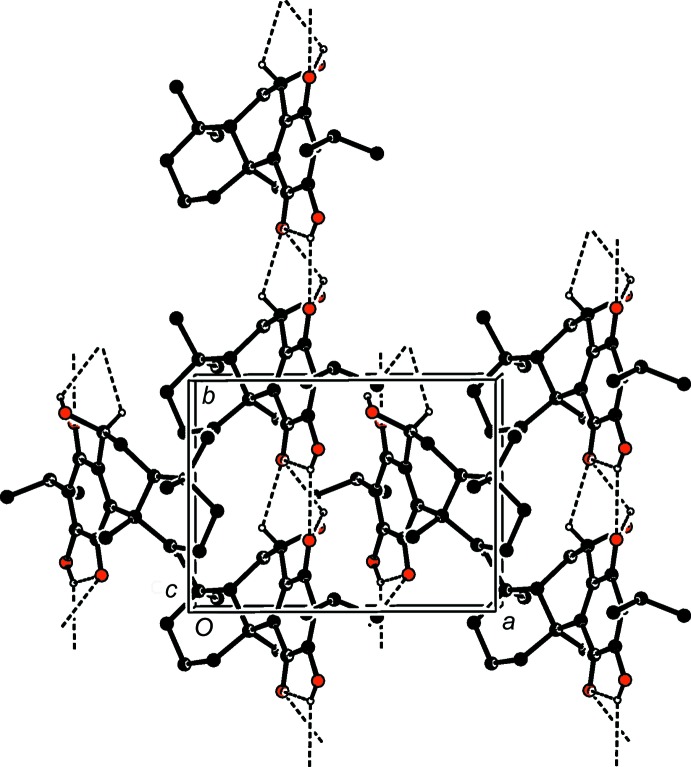
A view along the *c* axis of the crystal packing of the title compound, with hydrogen bonds shown as dashed lines. Only H atoms involved in these inter­actions have been included.

In the crystal, two strong O—H⋯O hydrogen bonds, namely O2—H2*A*⋯O3^i^ and O4—H4⋯O1^ii^, both approximately running along the *b* axis, are formed *via* the hy­droxy group and the carbonyl groups (Fig. 2 ▸ and Table 1 ▸). Furthermore, a weak C11—H11⋯O1^ii^ hydrogen bond occurs from a ring C atom to a carbonyl group, also running along the *b*-axis direction. These inter­actions result in the formation of chains propagating along the *b*-axis direction (Fig. 2 ▸ and Table 1 ▸).

## Database survey   

A search of the Cambridge Structural Database (CSD, Version 5.27, last update February 2017; Groom *et al.*, 2016 ▸) for the octa­hydro­phenanthrene-1,4-dione skeleton revealed 14 entries. These include two reports of a compound similar to the title compound, but with no hy­droxy group in position 10, *i.e.* CSD refcodes HACGUN (Eugster *et al.*, 1993 ▸) and HACGUN01 (Fun *et al.*, 2011 ▸), and two reports of the stereoisomer of the title compound, with the hy­droxy group in position 10 having an *R* configuration, *i.e.* QICLIX (Chen *et al.*, 2000 ▸) and QICLIX01 (Razak *et al.*, 2010 ▸).

## Isolation and crystallization   

The title compound was isolated from the pollen of *Taxodium ascendens*, collected in Wuhan, China, in April 2013 (SC0123). The air-dried pollen (1.8 kg) was extracted with 95% ethanol and then partitioned successively with petroleum ether (PE), ethyl acetate (EtOAc) and *n*-butyl alcohol (*n*-BuOH) to give a PE extract (80 g), an EtOAc extract (120 g) and a *n*-BuOH extract (100 g). The PE extract (80 g) was subjected to normal-phase silica-gel column chromatography (300–400 mesh) with a gradient solvent system of petroleum ether–acetone (1.0–0.1 *v*/*v*, containing 0.1% formic acid) to give eight major fractions, denoted F1–F8. Fraction F4 (6 g) was sequentially subjected to normal-phase silica-gel column chromatography (300–400 mesh) with an isocratic elution (petroleum ether–acetone, 2:1 *v*/*v*, containing 0.1% formic acid) to give three major fractions, denoted F4.1, F4.2 and F4.3. Fraction F4.3 was purified by semipreparative HPLC (CNCH_3_/H_2_O, 10:90→100:0, 40 min, containing 0.1% formic acid in both phase), to give an orange solid, which was recrystallized from the mixed solvents of CH_2_Cl_2_–MeOH (5:2 *v*/*v*), affording orange block-like crystals suitable for X-ray diffraction analysis. The ^1^H and ^13^C NMR data of 7β-hy­droxy­royleanone have been reported elsewhere (Chang & Zhu, 2001 ▸).

## Refinement   

Crystal data, data collection and structure refinement details are summarized in Table 2 ▸. The H atoms were positioned with idealized geometry and refined using a riding model, with O—H = 0.82 Å and C—H = 0.94–0.98 Å, and with *U*_iso_(H) = 1.5*U*_eq_(O,C) for hydroxy and methyl groups, and 1.2*U*_eq_(C) for other H atoms.

## Supplementary Material

Crystal structure: contains datablock(s) I, Global. DOI: 10.1107/S2056989017011987/su5382sup1.cif

Structure factors: contains datablock(s) I. DOI: 10.1107/S2056989017011987/su5382Isup2.hkl

Supporting information file. DOI: 10.1107/S2056989017011987/su5382Isup3.cdx

Supporting information file. DOI: 10.1107/S2056989017011987/su5382Isup4.cml

CCDC reference: 1551129

Additional supporting information:  crystallographic information; 3D view; checkCIF report

## Figures and Tables

**Table 1:** Hydrogen-bond geometry (Å, °)

*D*—H⋯*A*	*D*—H	H⋯*A*	*D*⋯*A*	*D*—H⋯*A*
O2—H2*A*⋯O1	0.82	2.10	2.579 (2)	117
O4—H4⋯O3	0.82	2.37	2.814 (2)	115
O2—H2*A*⋯O3^i^	0.82	2.39	3.153 (2)	155
O4—H4⋯O1^ii^	0.82	2.33	2.901 (2)	127
C11—H11⋯O1^ii^	0.98	2.47	3.120 (2)	124

Symmetry codes: (i) 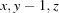; (ii) 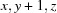.

**Table 2:** Experimental details

Crystal data	
Chemical formula	C_20_H_28_O_4_
*M*_r_	332.42
Crystal system, space group	Monoclinic, *P*2_1_
Temperature (K)	296
*a*, *b*, *c* (Å)	10.2570 (18), 7.6151 (13), 11.503 (2)
β (°)	101.110 (3)
*V* (Å^3^)	881.6 (3)
*Z*	2
Radiation type	Mo *K*α
μ (mm^−1^)	0.09
Crystal size (mm)	0.15 × 0.12 × 0.10

Data collection	
Diffractometer	Bruker APEXII CCD
No. of measured, independent and observed [*I* > 2σ(*I*)] reflections	6669, 3463, 3163
*R*_int_	0.024
(sin θ/λ)_max_ (Å^−1^)	0.617

Refinement	
*R*[*F* ^2^ > 2σ(*F* ^2^)], *wR*(*F* ^2^), *S*	0.030, 0.083, 1.03
No. of reflections	3319
No. of parameters	225
No. of restraints	1
H-atom treatment	H-atom parameters constrained
Δρ_max_, Δρ_min_ (e Å^−3^)	0.18, −0.11
Absolute structure	Flack *x* determined using 1341 quotients [(*I* ^+^) − (*I* ^−^)]/[(*I* ^+^) + (*I* ^−^)] (Parsons *et al.*, 2013 ▸)
Absolute structure parameter	0.2 (3)

Computer programs: *APEX2* (Bruker, 2007 ▸), *SAINT* (Bruker, 2007 ▸), *SHELXS97* (Sheldrick, 2008 ▸), *SHELXTL* (Sheldrick, 2008 ▸), *SHELXL2014* (Sheldrick, 2015 ▸) and *PLATON* (Spek, 2009 ▸).

## supplementary crystallographic information

### Crystal data

**Table:**

C_20_H_28_O_4_	*F*(000) = 360
*M**_r_* = 332.42	*D*_x_ = 1.252 Mg m^−^^3^
Monoclinic, *P*2_1_	Mo *K*α radiation, λ = 0.71073 Å
*a* = 10.2570 (18) Å	Cell parameters from 5396 reflections
*b* = 7.6151 (13) Å	θ = 2.4–31.8°
*c* = 11.503 (2) Å	µ = 0.09 mm^−^^1^
β = 101.110 (3)°	*T* = 296 K
*V* = 881.6 (3) Å^3^	Block, orange
*Z* = 2	0.15 × 0.12 × 0.10 mm

### Data collection

**Table:**

Bruker APEXII CCD diffractometer	*R*_int_ = 0.024
φ and ω scans	θ_max_ = 26.0°, θ_min_ = 1.8°
6669 measured reflections	*h* = −12→12
3463 independent reflections	*k* = −9→8
3163 reflections with *I* > 2σ(*I*)	*l* = −14→14

### Refinement

**Table:**

Refinement on *F*^2^	Hydrogen site location: inferred from neighbouring sites
Least-squares matrix: full	H-atom parameters constrained
*R*[*F*^2^ > 2σ(*F*^2^)] = 0.030	*w* = 1/[σ^2^(*F*_o_^2^) + (0.0415*P*)^2^ + 0.1205*P*] where *P* = (*F*_o_^2^ + 2*F*_c_^2^)/3
*wR*(*F*^2^) = 0.083	(Δ/σ)_max_ = 0.018
*S* = 1.03	Δρ_max_ = 0.18 e Å^−^^3^
3319 reflections	Δρ_min_ = −0.11 e Å^−^^3^
225 parameters	Extinction correction: (SHELXL2014; Sheldrick, 2015), Fc^*^=kFc[1+0.001xFc^2^λ^3^/sin(2θ)]^-1/4^
1 restraint	Extinction coefficient: 0.042 (5)
Primary atom site location: structure-invariant direct methods	Absolute structure: Flack *x* determined using 1341 quotients [(I+)-(I-)]/[(I+)+(I-)] (Parsons *et al.*, 2013)
Secondary atom site location: difference Fourier map	Absolute structure parameter: 0.2 (3)

### Special details

**Table:**

Geometry. All esds (except the esd in the dihedral angle between two l.s. planes) are estimated using the full covariance matrix. The cell esds are taken into account individually in the estimation of esds in distances, angles and torsion angles; correlations between esds in cell parameters are only used when they are defined by crystal symmetry. An approximate (isotropic) treatment of cell esds is used for estimating esds involving l.s. planes.

### Fractional atomic coordinates and isotropic or equivalent isotropic displacement parameters (Å^2^)

**Table:**

	*x*	*y*	*z*	*U*_iso_*/*U*_eq_	
C1	0.18336 (18)	0.9032 (3)	0.38131 (16)	0.0325 (4)	
C2	0.12043 (19)	1.0797 (3)	0.33060 (16)	0.0330 (4)	
H2	0.0708	1.1210	0.3901	0.040*	
C3	0.0151 (2)	1.0710 (3)	0.21358 (18)	0.0436 (5)	
C4	−0.0879 (2)	0.9310 (4)	0.2287 (2)	0.0558 (6)	
H4A	−0.1416	0.9755	0.2830	0.067*	
H4B	−0.1464	0.9124	0.1527	0.067*	
C5	−0.0293 (2)	0.7561 (3)	0.2744 (2)	0.0552 (6)	
H5A	0.0162	0.7042	0.2163	0.066*	
H5B	−0.1003	0.6773	0.2852	0.066*	
C6	0.0680 (2)	0.7766 (3)	0.3915 (2)	0.0449 (5)	
H6A	0.1043	0.6625	0.4175	0.054*	
H6B	0.0210	0.8209	0.4509	0.054*	
C7	0.2771 (2)	0.8148 (3)	0.30881 (19)	0.0468 (5)	
H7A	0.3432	0.8976	0.2954	0.070*	
H7B	0.2267	0.7760	0.2341	0.070*	
H7C	0.3198	0.7158	0.3519	0.070*	
C8	0.0733 (3)	1.0359 (5)	0.1025 (2)	0.0652 (7)	
H8A	0.1034	0.9164	0.1032	0.098*	
H8B	0.1467	1.1137	0.1015	0.098*	
H8C	0.0061	1.0555	0.0331	0.098*	
C9	−0.0575 (3)	1.2484 (4)	0.1956 (3)	0.0632 (7)	
H9A	0.0009	1.3358	0.1739	0.095*	
H9B	−0.0841	1.2826	0.2679	0.095*	
H9C	−0.1347	1.2376	0.1338	0.095*	
C10	0.2287 (2)	1.2171 (3)	0.33153 (17)	0.0393 (4)	
H10A	0.2976	1.1703	0.2932	0.047*	
H10B	0.1914	1.3204	0.2881	0.047*	
C11	0.28762 (18)	1.2668 (2)	0.45820 (17)	0.0329 (4)	
H11	0.2280	1.3501	0.4866	0.040*	
C12	0.30669 (18)	1.1084 (2)	0.53977 (16)	0.0304 (4)	
C13	0.26614 (18)	0.9456 (2)	0.50399 (16)	0.0304 (4)	
C14	0.3121 (2)	0.8004 (3)	0.58809 (17)	0.0346 (4)	
C15	0.38257 (19)	0.8399 (3)	0.71053 (17)	0.0354 (4)	
C16	0.40798 (19)	1.0040 (3)	0.75146 (17)	0.0347 (4)	
C17	0.36998 (17)	1.1471 (3)	0.66582 (17)	0.0319 (4)	
C18	0.4710 (2)	1.0503 (3)	0.87781 (17)	0.0434 (5)	
H18	0.4779	1.1785	0.8825	0.052*	
C19	0.3826 (3)	0.9924 (4)	0.9633 (2)	0.0596 (7)	
H19A	0.2956	1.0422	0.9390	0.089*	
H19B	0.4203	1.0321	1.0418	0.089*	
H19C	0.3761	0.8667	0.9629	0.089*	
C20	0.6116 (2)	0.9766 (4)	0.9132 (2)	0.0596 (7)	
H20A	0.6080	0.8506	0.9140	0.089*	
H20B	0.6511	1.0186	0.9907	0.089*	
H20C	0.6640	1.0143	0.8571	0.089*	
O1	0.29606 (19)	0.6460 (2)	0.56208 (14)	0.0538 (4)	
O2	0.41656 (17)	0.6964 (2)	0.77776 (13)	0.0485 (4)	
H2A	0.3912	0.6082	0.7391	0.073*	
O3	0.38668 (15)	1.30061 (19)	0.69666 (13)	0.0441 (4)	
O4	0.41202 (14)	1.3502 (2)	0.45960 (14)	0.0458 (4)	
H4	0.4286	1.4170	0.5165	0.069*	

### Atomic displacement parameters (Å^2^)

**Table:**

	*U*^11^	*U*^22^	*U*^33^	*U*^12^	*U*^13^	*U*^23^
C1	0.0336 (9)	0.0310 (10)	0.0314 (9)	0.0013 (7)	0.0025 (7)	−0.0020 (7)
C2	0.0350 (9)	0.0332 (10)	0.0304 (8)	0.0038 (8)	0.0052 (7)	0.0013 (7)
C3	0.0406 (11)	0.0488 (13)	0.0373 (10)	0.0060 (9)	−0.0025 (8)	0.0032 (9)
C4	0.0404 (11)	0.0618 (17)	0.0577 (14)	−0.0007 (11)	−0.0090 (10)	0.0018 (12)
C5	0.0486 (13)	0.0476 (15)	0.0628 (14)	−0.0103 (10)	−0.0057 (11)	−0.0022 (11)
C6	0.0455 (11)	0.0353 (12)	0.0499 (12)	−0.0063 (9)	−0.0008 (9)	0.0018 (9)
C7	0.0472 (12)	0.0483 (14)	0.0435 (11)	0.0137 (10)	0.0048 (9)	−0.0089 (10)
C8	0.0711 (16)	0.087 (2)	0.0342 (11)	0.0040 (15)	0.0019 (10)	−0.0009 (13)
C9	0.0588 (15)	0.0589 (18)	0.0639 (15)	0.0147 (12)	−0.0083 (12)	0.0115 (13)
C10	0.0454 (11)	0.0375 (11)	0.0343 (9)	−0.0008 (9)	0.0059 (8)	0.0086 (9)
C11	0.0348 (9)	0.0251 (10)	0.0386 (9)	0.0002 (7)	0.0062 (7)	0.0025 (7)
C12	0.0296 (8)	0.0283 (10)	0.0331 (9)	0.0036 (7)	0.0057 (7)	0.0015 (7)
C13	0.0319 (9)	0.0274 (10)	0.0312 (9)	0.0035 (7)	0.0042 (7)	−0.0006 (7)
C14	0.0409 (10)	0.0244 (10)	0.0370 (9)	0.0001 (8)	0.0038 (7)	0.0002 (8)
C15	0.0421 (10)	0.0280 (10)	0.0343 (9)	0.0038 (8)	0.0030 (8)	0.0053 (7)
C16	0.0376 (9)	0.0330 (11)	0.0315 (9)	−0.0003 (8)	0.0017 (7)	0.0000 (8)
C17	0.0303 (8)	0.0276 (10)	0.0374 (9)	0.0008 (7)	0.0058 (7)	−0.0008 (8)
C18	0.0539 (12)	0.0355 (11)	0.0351 (10)	−0.0038 (9)	−0.0058 (8)	−0.0008 (9)
C19	0.0685 (16)	0.0722 (19)	0.0364 (11)	−0.0044 (13)	0.0059 (10)	−0.0088 (11)
C20	0.0518 (13)	0.0643 (17)	0.0538 (13)	−0.0058 (12)	−0.0119 (11)	0.0043 (12)
O1	0.0789 (11)	0.0247 (8)	0.0491 (9)	0.0005 (8)	−0.0098 (8)	−0.0004 (7)
O2	0.0687 (10)	0.0291 (8)	0.0405 (8)	0.0007 (7)	−0.0074 (7)	0.0059 (6)
O3	0.0557 (9)	0.0269 (8)	0.0456 (8)	−0.0017 (6)	−0.0004 (7)	−0.0048 (6)
O4	0.0443 (8)	0.0421 (9)	0.0517 (8)	−0.0115 (7)	0.0111 (6)	0.0015 (7)

### Geometric parameters (Å, º)

**Table:**

C1—C13	1.535 (2)	C10—C11	1.514 (3)
C1—C7	1.543 (3)	C10—H10A	0.9700
C1—C6	1.547 (3)	C10—H10B	0.9700
C1—C2	1.555 (3)	C11—O4	1.423 (2)
C2—C10	1.525 (3)	C11—C12	1.517 (3)
C2—C3	1.556 (3)	C11—H11	0.9800
C2—H2	0.9800	C12—C13	1.347 (3)
C3—C4	1.534 (4)	C12—C17	1.499 (3)
C3—C8	1.534 (3)	C13—C14	1.485 (3)
C3—C9	1.538 (4)	C14—O1	1.217 (3)
C4—C5	1.514 (4)	C14—C15	1.485 (3)
C4—H4A	0.9700	C15—C16	1.343 (3)
C4—H4B	0.9700	C15—O2	1.345 (2)
C5—C6	1.522 (3)	C16—C17	1.470 (3)
C5—H5A	0.9700	C16—C18	1.514 (3)
C5—H5B	0.9700	C17—O3	1.224 (2)
C6—H6A	0.9700	C18—C19	1.526 (3)
C6—H6B	0.9700	C18—C20	1.527 (3)
C7—H7A	0.9600	C18—H18	0.9800
C7—H7B	0.9600	C19—H19A	0.9600
C7—H7C	0.9600	C19—H19B	0.9600
C8—H8A	0.9600	C19—H19C	0.9600
C8—H8B	0.9600	C20—H20A	0.9600
C8—H8C	0.9600	C20—H20B	0.9600
C9—H9A	0.9600	C20—H20C	0.9600
C9—H9B	0.9600	O2—H2A	0.8200
C9—H9C	0.9600	O4—H4	0.8200
			
C13—C1—C7	107.26 (15)	H9A—C9—H9C	109.5
C13—C1—C6	110.93 (16)	H9B—C9—H9C	109.5
C7—C1—C6	109.52 (18)	C11—C10—C2	109.46 (15)
C13—C1—C2	106.21 (15)	C11—C10—H10A	109.8
C7—C1—C2	115.53 (17)	C2—C10—H10A	109.8
C6—C1—C2	107.36 (15)	C11—C10—H10B	109.8
C10—C2—C1	109.97 (15)	C2—C10—H10B	109.8
C10—C2—C3	114.73 (16)	H10A—C10—H10B	108.2
C1—C2—C3	117.15 (16)	O4—C11—C10	108.25 (15)
C10—C2—H2	104.5	O4—C11—C12	109.85 (15)
C1—C2—H2	104.5	C10—C11—C12	112.14 (16)
C3—C2—H2	104.5	O4—C11—H11	108.9
C4—C3—C8	111.1 (2)	C10—C11—H11	108.9
C4—C3—C9	107.4 (2)	C12—C11—H11	108.8
C8—C3—C9	107.3 (2)	C13—C12—C17	121.81 (17)
C4—C3—C2	108.08 (18)	C13—C12—C11	123.18 (16)
C8—C3—C2	114.31 (18)	C17—C12—C11	114.97 (16)
C9—C3—C2	108.44 (19)	C12—C13—C14	116.48 (16)
C5—C4—C3	114.45 (19)	C12—C13—C1	123.93 (17)
C5—C4—H4A	108.6	C14—C13—C1	119.50 (16)
C3—C4—H4A	108.6	O1—C14—C13	123.26 (19)
C5—C4—H4B	108.6	O1—C14—C15	116.57 (18)
C3—C4—H4B	108.6	C13—C14—C15	120.16 (17)
H4A—C4—H4B	107.6	C16—C15—O2	122.88 (17)
C4—C5—C6	111.5 (2)	C16—C15—C14	123.19 (18)
C4—C5—H5A	109.3	O2—C15—C14	113.92 (17)
C6—C5—H5A	109.3	C15—C16—C17	116.50 (17)
C4—C5—H5B	109.3	C15—C16—C18	124.83 (19)
C6—C5—H5B	109.3	C17—C16—C18	118.67 (18)
H5A—C5—H5B	108.0	O3—C17—C16	120.63 (18)
C5—C6—C1	112.19 (18)	O3—C17—C12	118.58 (17)
C5—C6—H6A	109.2	C16—C17—C12	120.78 (17)
C1—C6—H6A	109.2	C16—C18—C19	110.77 (18)
C5—C6—H6B	109.2	C16—C18—C20	112.1 (2)
C1—C6—H6B	109.2	C19—C18—C20	111.7 (2)
H6A—C6—H6B	107.9	C16—C18—H18	107.3
C1—C7—H7A	109.5	C19—C18—H18	107.3
C1—C7—H7B	109.5	C20—C18—H18	107.3
H7A—C7—H7B	109.5	C18—C19—H19A	109.5
C1—C7—H7C	109.5	C18—C19—H19B	109.5
H7A—C7—H7C	109.5	H19A—C19—H19B	109.5
H7B—C7—H7C	109.5	C18—C19—H19C	109.5
C3—C8—H8A	109.5	H19A—C19—H19C	109.5
C3—C8—H8B	109.5	H19B—C19—H19C	109.5
H8A—C8—H8B	109.5	C18—C20—H20A	109.5
C3—C8—H8C	109.5	C18—C20—H20B	109.5
H8A—C8—H8C	109.5	H20A—C20—H20B	109.5
H8B—C8—H8C	109.5	C18—C20—H20C	109.5
C3—C9—H9A	109.5	H20A—C20—H20C	109.5
C3—C9—H9B	109.5	H20B—C20—H20C	109.5
H9A—C9—H9B	109.5	C15—O2—H2A	109.5
C3—C9—H9C	109.5	C11—O4—H4	109.5
			
C13—C1—C2—C10	54.86 (19)	C11—C12—C13—C1	−6.7 (3)
C7—C1—C2—C10	−63.9 (2)	C7—C1—C13—C12	105.6 (2)
C6—C1—C2—C10	173.59 (16)	C6—C1—C13—C12	−134.8 (2)
C13—C1—C2—C3	−171.78 (16)	C2—C1—C13—C12	−18.5 (2)
C7—C1—C2—C3	69.5 (2)	C7—C1—C13—C14	−70.9 (2)
C6—C1—C2—C3	−53.1 (2)	C6—C1—C13—C14	48.6 (2)
C10—C2—C3—C4	−178.36 (19)	C2—C1—C13—C14	164.98 (16)
C1—C2—C3—C4	50.4 (2)	C12—C13—C14—O1	−171.2 (2)
C10—C2—C3—C8	57.4 (3)	C1—C13—C14—O1	5.6 (3)
C1—C2—C3—C8	−73.8 (3)	C12—C13—C14—C15	8.3 (3)
C10—C2—C3—C9	−62.3 (2)	C1—C13—C14—C15	−174.92 (17)
C1—C2—C3—C9	166.5 (2)	O1—C14—C15—C16	179.6 (2)
C8—C3—C4—C5	76.0 (3)	C13—C14—C15—C16	0.1 (3)
C9—C3—C4—C5	−166.9 (2)	O1—C14—C15—O2	−1.6 (3)
C2—C3—C4—C5	−50.1 (3)	C13—C14—C15—O2	178.92 (17)
C3—C4—C5—C6	56.2 (3)	O2—C15—C16—C17	177.42 (18)
C4—C5—C6—C1	−58.2 (3)	C14—C15—C16—C17	−3.9 (3)
C13—C1—C6—C5	170.39 (19)	O2—C15—C16—C18	−3.1 (3)
C7—C1—C6—C5	−71.4 (2)	C14—C15—C16—C18	175.61 (19)
C2—C1—C6—C5	54.8 (2)	C15—C16—C17—O3	178.2 (2)
C1—C2—C10—C11	−68.9 (2)	C18—C16—C17—O3	−1.4 (3)
C3—C2—C10—C11	156.51 (17)	C15—C16—C17—C12	−0.2 (3)
C2—C10—C11—O4	162.26 (16)	C18—C16—C17—C12	−179.73 (17)
C2—C10—C11—C12	40.9 (2)	C13—C12—C17—O3	−169.35 (19)
O4—C11—C12—C13	−125.08 (19)	C11—C12—C17—O3	8.4 (2)
C10—C11—C12—C13	−4.7 (3)	C13—C12—C17—C16	9.0 (3)
O4—C11—C12—C17	57.2 (2)	C11—C12—C17—C16	−173.16 (16)
C10—C11—C12—C17	177.58 (16)	C15—C16—C18—C19	−63.3 (3)
C17—C12—C13—C14	−12.5 (3)	C17—C16—C18—C19	116.2 (2)
C11—C12—C13—C14	169.93 (16)	C15—C16—C18—C20	62.2 (3)
C17—C12—C13—C1	170.90 (16)	C17—C16—C18—C20	−118.2 (2)

### Hydrogen-bond geometry (Å, º)

**Table:**

*D*—H···*A*	*D*—H	H···*A*	*D*···*A*	*D*—H···*A*
O2—H2*A*···O1	0.82	2.10	2.579 (2)	117
O4—H4···O3	0.82	2.37	2.814 (2)	115
O2—H2*A*···O3^i^	0.82	2.39	3.153 (2)	155
O4—H4···O1^ii^	0.82	2.33	2.901 (2)	127
C11—H11···O1^ii^	0.98	2.47	3.120 (2)	124

Symmetry codes: (i) *x*, *y*−1, *z*; (ii) *x*, *y*+1, *z*.
